# Prediction of bronchodilation test in adults with chronic cough suspected of cough variant asthma

**DOI:** 10.3389/fmed.2022.987887

**Published:** 2022-12-09

**Authors:** Huijuan Hao, Yilin Pan, Zichong Xu, Zengchao Xu, Wuping Bao, Yishu Xue, Chengjian Lv, Jingwang Lin, Yingying Zhang, Min Zhang

**Affiliations:** ^1^Department of Respiratory and Critical Care Medicine, Shanghai General Hospital, Shanghai Jiao Tong University School of Medicine, Shanghai, China; ^2^Department of Mathematics, Shanghai Normal University, Shanghai, China

**Keywords:** cough variant asthma (CVA), bronchodilation test, forced expiratory volume in 1 s (FEV1), fractional exhaled nitric oxide (FENO), diagnosis

## Abstract

**Background:**

Many patients with cough variant asthma (CVA) are underdiagnosed and undertreated due to the atypical symptoms, low diagnostic sensitivity of bronchodilator response (BDR), and limited application of bronchial challenge test.

**Objective:**

To investigate whether airway reversibility in BDR can predict CVA diagnosis in patients with chronic cough and negative BDR.

**Methods:**

This open-label, prospective cohort study included patients with chronic cough, nearly normal chest CT scan, and negative BDR results. Inhaled corticosteroids and long-acting β_2_ agonists were given for 4 weeks. The confirmed diagnosis of CVA was defined as improved symptoms and an increase of forced expiratory volume in 1 s (FEV_1_) by >12% and >200 mL after 4 weeks of treatment.

**Results:**

Of 155 patients recruited, 140 completed the study. Patients in the CVA positive diagnosis group had greater absolute (Δ) and percent (Δ%) improvements in FEV_1_ and forced expiratory flows (FEFs), and higher fractional exhaled nitric oxide (FENO) than in the CVA negative diagnosis group. The area under the receiver operating characteristic curves (AUCs) of ΔFEV_1_%, FEF_25–75_%pred (percentage of predicted forced expiratory flow at 25% to 75%) and FENO for CVA positive diagnosis was 0.825, 0.714, and 0.637, with cutoff values of 5.90%, 61.99% and 41.50 ppb, respectively. A joint model of ΔFEV_1_% combined with FEF_25–75_%pred or FENO increased the AUC to 0.848 and 0.847, respectively.

**Conclusion:**

ΔFEV_1_% in BDR can predict a CVA diagnosis and response to anti-asthma treatment in patients with chronic cough and negative BDR.

**Clinical trial registration:**

[http://www.chictr.org.cn/index.aspx], identifier [ChiCTR2000029065].

## Introduction

Cough variant asthma (CVA), with a sole or main symptom of cough, is a special phenotype of asthma and one of the most common causes of chronic cough. In China, CVA accounts for more than one-third of the causes of chronic cough (1, 2). The hallmarks of CVA include bronchial hyperresponsiveness (BHR) and successful treatment with inhaled corticosteroids (ICS) and/or bronchodilators (3).

Despite atypical symptoms and high levels of heterogeneity among CVA patients, some patients develop classic asthma over time (4–6). On top of that, severe cough can be highly disruptive to individual life, leaving patients vulnerable to a variety of comorbidities such as incontinence, cough syncope, dysphonia, depression, and difficulties in relationships (7). Early detection and treatment with ICS prove crucial to those patients as it aids symptom control and may prevent the progression of CVA to classic asthma (4). However, given the atypical symptoms and near-normal forced expiratory volume in 1 s (FEV_1_) in spirometry, the precise diagnosis of CVA remains very challenging, especially for primary care physicians. The European and American guidelines recommend that the CVA diagnosis should be determined according to the documentation of variability in lung function and the therapeutic response (3, 8).

Objective indicators of CVA diagnosis require evidence of confirmed variable airway limitation defined by either a positive bronchodilator response (BDR), or BHR defined by a positive bronchial challenge test (BCT). Generally, the BCT is recommended for diagnosing CVA. However, in light of the costly, time-consuming process, requirements of professional technicians and equipment (9), as well as the potential risk of severe bronchospasm (10), it remains difficult to be appropriately carried out, especially in primary care settings. On the other hand, although BDR is safer, more convenient and widespread-used, and with a higher specificity, its sensitivity for CVA is low because a 12% improvement of FEV_1_ is difficult to achieve, especially among those patients with nearly normal baseline FEV_1_. Unfortunately, most CVA patients prefer primary care settings for their relatively mild and atypical symptoms, thus putting appropriate diagnosis and treatment of CVA in a dilemma due to the shortage of effective detection methods.

An increase in FEV_1_ by >12% and >200 ml from baseline after 4 weeks of anti-asthma treatment is also recommended as a diagnostic criterion of asthma in GINA (8), which can be considered a positive BDR after anti-asthma treatment (BAAT). However, the guidelines do not provide specific descriptions as to which group of potential CVA patients may benefit most from this diagnostic therapy. Additionally, primary care physicians tend to be reluctant to prescribe inhaled corticosteroids due to potential side effects, and instead, they prefer to prescribe antibiotics and antitussive drugs. Therefore, it would be very helpful if a more convenient and efficient method is developed, which aims to identify those CVA patients who can potentially benefit most from the diagnostic therapy at baseline.

Based on those facts, the purpose of our research was to investigate whether spirometric indices change alone or in their different combinations, could predict patients’ responses to anti-asthma treatment, and therefore help physicians prescribe ICS precisely and improve the diagnostic rate of CVA in patients with chronic cough and negative BDR.

## Materials and methods

### Study design and participants

This study protocol was approved by the Institutional Review Board at Shanghai General Hospital (no. [2020]30) and registered on http://www.chictr.org.cn/index.aspx (No. ChiCTR2000029065). From April 1, 2020, to January 30, 2021, participants were consecutively recruited via the Pulmonary Outpatient Clinic of Shanghai General Hospital (Shanghai, China). To be included in this study, participants must have been aged 18–65 years with a sole or predominant symptom of chronic cough, and the spirometry satisfied the criteria that FEV_1_/forced vital capacity (FVC) was more than 0.7 after administration of salbutamol, BDR was negative according to the GINA standards.

The exclusion criteria included a respiratory infection within 8 weeks before screening and cigarette smoking including current smoking, cessation within 2 months, and a smoking history of more than 10 pack-years. Patients with the symptom of gastroesophageal reflux disease and upper airway cough syndrome were also excluded. All patients underwent a visual analog scale score (VAS) of cough to assess the severity of symptoms, a fractional exhaled nitric oxide (FENO) and a spirometry to assess the airway inflammation and constriction, and a blood cell counts, an echocardiography, and a high-resolution computed tomography scan to exclude concomitant systemic respiratory and cardiac disease. The usage of drugs that may affect the spirometry results and lead to chronic cough was also an important exclusion criterion.

Enrolled patients were given 4 weeks of ICS and long-acting β_2_-agonist (LABA) treatment (Symbicort Turbuhaler, 160 mg budesonide and 4.5 mg formoterol per dose, 1 dose twice per day, AstraZeneca). Considering the circadian rhythms, after 4 weeks of ICS/LABA treatment, the follow-up spirometry and VAS were performed at the same time of the first visit (8 am–10 am) and allowed 2 days of adjustment before or after the scheduled date, according to the actual situation of the subjects. Symptom recovery time was assessed weekly by telephone or WeChat and defined as the time from the start of treatment to symptom improvement, and the symptom improvement was judged by a decrease in the VAS (ΔVAS = VAS_1_–VAS_2_ > 30 mm) (11).

### Spirometry, bronchodilation test, and fractional exhaled nitric oxide measurements

Spirometry and BDR were performed by the same technologist with the same spirometer (Jaeger Co., Hochberg, Germany) following the specifications and performance criteria recommended in the American Thoracic Society/European Respiratory Society Standardization (12). Participants underwent spirometry before and 15 min after inhaling salbutamol (400ug, Ventolin, salbutamol sulfate inhaled aerosol, Registration ID: JX20080307, 400 mg, GlaxoSmithKline).

The response to the bronchodilator was expressed as the percentage change relative to the prebronchodilator value of FEV_1_ (ΔFEV_1_%), FVC (ΔFVC%), and forced expiratory flows (FEFs; ΔFEFs%) and as the absolute change of ΔFEV_1_, ΔFVC, and ΔFEFs. The response to the 4 weeks of anti-inflammation was also expressed as the percentage improvement relative to the baseline of improvement-FEV_1_%, improvement-FVC%, and improvement-FEFs% and as the absolute change of improvement-FEV_1_, improvement-FVC, and improvement-FEFs.

Fractional exhaled nitric oxide (FENO) (NIOX MINO, Aerocrine AB, Solna, Sweden) was performed before spirometry since the involved breathing maneuvers could distort FENO results.

### Group definition

After 4 weeks of therapy, patients were divided into the CVA positive diagnosis group and the CVA negative diagnosis group according to their improvement of FEV_1_ and symptoms: a positive diagnosis of CVA was defined as an increase in FEV_1_ by more than 12% and 200 ml, and ΔVAS greater than 30mm from baseline to after 4 weeks of therapy. In CVA negative diagnosis group, according to their improvement in FEV_1_ and symptoms: patients with an increase in FEV_1_ by less than 12% and more than 200 ml, and ΔVAS greater than 30mm from baseline to after 4 weeks of therapy were divided into a suspected group. Patients with an increase in FEV_1_ by less than 12% and 200 ml, regardless of the symptom changes, were divided into a negative group.

### Statistical analysis

Data analysis was performed with SPSS software version 23.0 (SPSS Inc, Chicago, Ill) and R studio version 4.0.2 (Window desktop, R packages “proc”). The normality of the data distribution was checked with the Kolmogorov-Smirnov test. Normally distributed data are presented as the mean ± SD. The independent *t*-test or Mann-Whitney test and Fisher exact test or chi-square test were performed for the analysis of intergroup differences for continuous variables and categorical variables, respectively. The differences among the three groups were analyzed with one-way analysis of variance (ANOVA) if normally distributed or by Kruskal-Wallis if not, and the differences between the two groups were analyzed with Student-Newman-Keuls.

The prediction performance of each variable was measured as the area under the curve (AUC) of the receiver-operating characteristic derived from the logistic regression models. The resultant AUC of multiple logistic models of the 2 variables was used as a measure of the joint prediction performance. The Delong test was used to determine whether the multiple logistic models would significantly improve the prediction performance (13). The threshold for statistical significance for all analyses was set at P less than 0.05.

## Results

### Demographic and clinical characteristics data

A total of 155 patients with chronic cough was enrolled, and 140 patients completed the 4 weeks of treatment and scheduled spirometry at the second visit; 9 patients were excluded because they did not attend the second visit on time, and 6 patients were excluded for taking medicine insufficient.

There were 98 patients in the CVA negative diagnosis group (70%), and 42 patients in the CVA positive diagnosis group (30%). All spirometric indices increased after bronchodilation for the two groups, and they increased further after the 4 weeks of treatment. FEV_1_%pred in most patients was more than 80%, only 16 (11%) patients were in 70%–80%, among whom 5 patients were in CVA negative diagnosis group and 11 patients were in CVA positive diagnosis group.

Most demographic data and eosinophils did not differ in the two groups at baseline (Table 1). However, all baseline spirometric indices including large airway indices (FVC%pred, FEV_1_%pred) and small airway indices (FEF_50_%pred, FEF_75_%pred, FEF_25–75_%pred) in the CVA positive diagnosis group were significantly lower than the CVA negative diagnosis group (*p* < 0.05). The improvement of spirometric in BDR including the percentage change (ΔFEV_1_%, ΔFVC%, ΔFEF_75_%, ΔFEF_25–75_%) and absolute change (ΔFEV_1_, ΔFVC, ΔFEF_75_, ΔFEF_25–75_) in the CVA positive diagnosis group was significantly higher than the CVA negative diagnosis group (*p* < 0.05). And the CVA positive diagnosis group had a higher baseline FENO (*p* = 0.010) and VAS score (*p* = 0.001). The symptom recovery time was longer in the CVA negative diagnosis group than in the CVA positive diagnosis group (*p* = 0.001) (Table 1).

**TABLE 1:** Demographic data and clinical features of participants in the CVA negative diagnosis group and CVA positive diagnosis group.

Characteristics and variables	CVA negative diagnosis group (*n* = 98)	CVA positive diagnosis group (*n* = 42)	*P*-value
Age(years)^¶^	45.50 [28.25]	42.00 [29.50]	0.622
Gender, male (n,%)	34 (34.69%)	12 (28.57%)	0.480
Height (cm)^¶^	162.50 [13.00]	165.00 [14.00]	0.980
Weight (kg)^¶^	60.00 [13.00]	62.00 [19.25]	0.539
BMI (kg/m^2^)^¶^	22.49 [3.70]	22.81 [4.72]	0.356
Former smoker (n,%)	11 (11.22%)	4 (9.52%)	0.766
Symptom duration (months)^¶^	7.00 [10.25]	9.50 [9.50]	0.221
Symptom recovery time (days)^¶^	**12.00 [15.25]**	**7.00 [5.50]**	**0.001**
VAS_1_^¶^	**75.00 [15.00]**	**80.00 [10.00]**	**0.001**
ΔVAS^¶^	**45.00 [47.50]**	**65.00 [16.30]**	**<0.001**
FENO (ppb)^¶^	**21.50 [22.25]**	**33.00 [32.25]**	**0.010**
WBC (*10^9^/L)^¶^	6.11 [2.49]	6.73 [2.06]	0.465
EOS%^¶^	1.90 [1.65]	1.95 [2.55]	0.742
EOS (*10^9^/L)^¶^	0.12 [0.12]	0.13 [0.20]	0.552
FVC%pred^§^	**100.20 ± 11.41**	**95.01 ± 13.34**	**0.021**
FEV_1_%pred^¶^	**97.00 [15.37]**	**87.05 [17.42]**	**<0.001**
FEV_1_/FVC	**0.82 ± 0.06**	**0.79 ± 0.05**	**0.021**
PEF%pred^§^	**97.00 ± 14.33**	**86.33 ± 18.68**	**<0.001**
FEF_25_%pred^§^	**95.77 ± 18.57**	**81.58 ± 18.00**	**<0.001**
FEF_50_%pred^§^	**80.10 ± 22.47**	**63.22 ± 14.19**	**<0.001**
FEF_75_%pred^¶^	**62.86 [32.98]**	**49.96 [33.97]**	**0.003**
FEF_25–75_%pred^§^	**75.54 ± 21.97**	**59.63 ± 16.91**	**<0.001**
ΔFVC^¶^	**50.00 [112.50]**	**120.00 [197.50]**	**0.001**
ΔFEV_1_^¶^	**100.00 [145.00]**	**185.00 [115.00]**	**<0.001**
ΔFEV_1_/FVC	0.02 [0.04]	0.03 [0.06]	0.039
ΔPEF^¶^	255.00 [780.00]	350.00 [880.00]	0.792
ΔFEF_25_^¶^	265.00 [817.50]	450.00 [1015.00]	0.168
ΔFEF_50_^¶^	340.00 [530.00]	525.00 [507.50]	0.127
ΔFEF_75_^¶^	**140.00 [280.00]**	**260.00 [250.00]**	**0.023**
ΔFEF_25–75_^¶^	**300.00 [395.00]**	**475.00 [397.50]**	**0.007**
ΔFVC%^¶^	**1.39 [3.87]**	**3.44 [7.69]**	**0.001**
ΔFEV_1_%^¶^	**3.77 [4.76]**	**7.99 [3.67]**	**<0.001**
ΔPEF%^¶^	3.89 [11.42]	6.45 [15.22]	0.588
ΔFEF_25_%^¶^	5.06 [13.96]	10.75 [21.65]	0.077
ΔFEF_50_%^§^	**12.94 ± 14.53**	**19.60 ± 18.87**	**0.025**
ΔFEF_75_%^¶^	**13.91 [31.98]**	**32.84 [43.87]**	**0.002**
ΔFEF_25–75_%^¶^	**12.63 [18.68]**	**23.24 [19.41]**	**<0.001**

FENO, fractional exhaled nitric oxide; VAS_1_, visual analog scale score at the first visit; ΔVAS, improvement of VAS from baseline to 4 weeks of treatment; WBC, white blood cells; EOS, eosinophils; BMI, body mass index; FVC, forced vital capacity; FEV_1_, forced expiratory volume in 1 s; PEF, peak expiratory flow; FEF_25_, forced expiratory flow at 25% of forced vital capacity; FEF_50_, forced expiratory flow at 50% of forced vital capacity; FEF_75_, forced expiratory flow at 75% of forced vital capacity; FEF_25–75_, forced expiratory flow at 25% to 75% of forced vital capacity; %pred, the actual measured value of spirometric indices as a percentage of the predicted value. Δ, increase in spirometric indices in BDR; Δ%, spirometric indices %, increase in spirometric indices as a percentage of the baseline value. ^§^Mean ± standard deviation values; ^¶^Median [IQR] values; Statistical significance is shown by bold font.

### Predictive values of a single measurement

The prognostic value of these variables for CVA diagnosis was calculated by AUC (Table 2). The largest AUC was ΔFEV_1_% (0.825, 95% CI 0.752 to 0.897) in BDR, with cutoff values of 5.90%. The AUCs of the small airway, such as FEF_50_%pred, FEF_75_%pred, and FEF_25–75_%pred for CVA diagnosis were 0.727, 0.659, and 0.714 with cutoff values of 72.48%, 55.31%, and 61.99%, respectively. The AUC of FENO for CVA diagnosis was 0.637 with a cutoff value of 41.50 ppb.

**TABLE 2:** Optimal cut-off values for the prediction of CVA positive diagnosis.

Characteristics and variables	Cutoff values*	AUC	Sensitivity %	Specificity %	PLR	NLR	PCC	PPV	NPV	*P*-value
FENO	41.50	0.637	38.10	83.67	2.33	0.74	70.00	50.00	75.93	0.005
FVC%pred	96.91	0.640	66.67	62.24	1.77	0.54	63.57	43.08	81.33	0.005
FEV_1_%pred	91.46	0.692	66.67	67.35	2.04	0.49	67.14	46.67	82.50	<0.001
PEF%pred	84.44	0.692	54.76	82.65	3.16	0.55	74.29	57.50	81.00	<0.001
FEF_25_%pred	83.71	0.727	69.05	74.49	2.71	0.42	72.86	53.70	84.88	<0.001
FEF_50_%pred	72.48	0.727	80.95	60.20	2.03	0.32	66.43	46.58	88.06	<0.001
FEF_75_%pred	55.31	0.659	61.90	67.35	1.90	0.57	65.71	44.83	80.49	0.002
FEF_25–75_%pred	61.99	0.714	61.90	74.49	2.43	0.51	70.71	50.98	82.02	<0.001
ΔFVC	170.00	0.673	42.86	90.82	4.67	0.63	76.43	66.67	78.76	0.001
ΔFEV_1_	120.00	0.748	88.10	59.18	2.16	0.20	67.86	48.05	92.06	<0.001
ΔFEF_75_	135.00	0.622	80.95	50.00	1.62	0.38	59.29	40.96	85.96	0.011
ΔFEF_25–75_	285.00	0.643	80.95	47.96	1.56	0.40	57.86	40.00	85.45	0.004
ΔFVC%	3.88	0.677	47.62	83.67	2.92	0.63	72.86	55.56	78.85	0.001
ΔFEV_1_%	5.90	0.825	83.33	72.45	3.02	0.23	75.71	56.45	91.03	<0.001
ΔFEF_50_%	17.75	0.626	59.52	70.41	2.01	0.57	67.14	46.30	80.23	0.009
ΔFEF_75_%	14.70	0.668	80.95	51.02	1.65	0.37	60.00	41.46	86.21	0.001
ΔFEF_25–75_%	15.16	0.699	80.95	56.12	1.85	0.34	63.57	44.16	87.30	<0.001

AUC, area under the curve; PLR, positive likelihood ratios; NLR, negative likelihood ratios; PPV, positive predictive value; NPV, negative predictive value; PCC, percentage correctly classified; *P*-value, the p value of logistic regression test. *The cut-off points were selected by maximizing the sum of sensitivity and specificity; The other abbreviations are as defined in Table 1.

### Predictive value of joint models: ΔFEV_1_% and fractional exhaled nitric oxide, ΔFEF_25–75_%, or FEF_25–75_%pred

**FIGURE 1:**
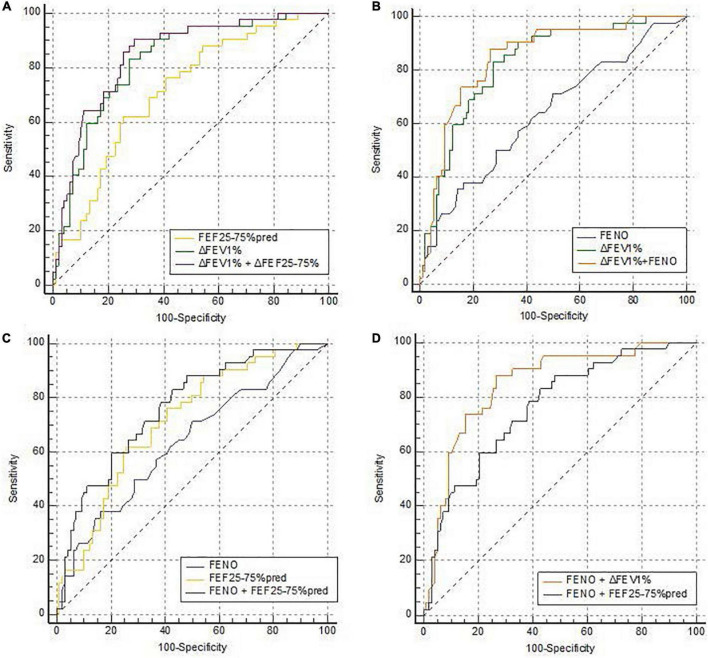
ROC curves of the joint models predicting CVA diagnosis. ROC curves for the joint models of **(A)** ΔFEV_1_% and FENO, **(C)** FENO and FEF_25–75_%pred, **(B)** ΔFEV_1_% and FEF_25–75_%pred, and **(D)** compare the AUC of FENO + ΔFEV_1_% and FENO + FEF_25–75_%pred in predicting CVA. **(A)** *n* = 140; AUC_Δ*FEV1% + FENO*_ = 0.847 (95% CI, 0.778 to 0.916); AUC_*FENO*_ = 0.637 (95% CI, 0.535 to 0.739); AUC_Δ*FEV1%*_ = 0.825 (95% CI, 0.752 to 0.897). **(C)** *n* = 140; AUC_FENO+FEF25–75%pred_ = 0.762 (95% CI, 0.677 to 0.847); AUC_*FENO*_ = 0.703 (95% CI, 0.601 to 0.805); AUC_FEF25–75%pred_ = 0.714 (95% CI, 0.624 to 0.804). **(B)** n = 140; AUC _Δ*FEV1%+FEF25*–75%pred_ = 0.848 (95% CI, 0.779 to 0.916); AUC_Δ*FEV1%*_ = 0.825 (95% CI, 0.752 to 0.897); AUC_FEF25–75%pred_ = 0.714 (95% CI, 0.624 to 0.804). CVA, cough variant asthma; ΔFEV_1_%, the increase in forced expiratory in 1 s as a percentage of baseline value in a bronchodilation test; FEF_25–75_%pred, percentage of predicted forced expiratory flow at 25% to 75%; FENO, fractional exhaled nitric oxide; ROC, receiver operating characteristic; AUC, area under the curve.

In the evaluated joint models, the two highest AUCs were the combination of ΔFEV_1_% + FEF_25–75_%pred (0.848, 95% CI 0.779 to 0.916) (Figure 1A), and the combination of ΔFEV_1_% + FENO (0.847, 95% CI 0.778 to 0.916) (Figure 1B). But the AUCs of the two joint models were not significantly higher than the AUC of ΔFEV_1_% alone (*p* = 0.069 and 0.076, respectively) (Table 3). The combination of FENO + FEF_25–75_%pred can increase AUC to 0.762 (Figure 1C). The results also demonstrated that the AUC of ΔFEV_1_% + FENO was higher than FEF_25–75_%pred + FENO (*p* = 0.051) (Figure 1D).

**TABLE 3:** Predictive values of the different joint models for the prediction of CVA positive diagnosis.

Characteristics and variables	AUC	95% CI (AUC)	PCC	Sensitivity %	Specificity %	PPV	NPV	*P*-value
FENO + FEF_25–75_%pred	**0.762**	**[0.677, 0.847]**	**67.14**	**78.57**	**62.24**	**47.14**	**87.14**	**0.003**
FENO + ΔFEV_1_%	0.847	[0.778, 0.916]	77.86	88.10	73.47	58.73	93.51	0.076
FENO + ΔFEF_25–75_%	0.727	[0.631, 0.823]	77.86	50.00	89.80	67.74	80.73	0.160
FEF_25–75_%pred + ΔFEV_1_%	0.848	[0.779, 0.916]	76.43	90.48	70.41	56.72	94.52	0.069
ΔFEV_1_ + ΔFEV_1_%	0.837	[0.767, 0.907]	74.29	92.86	66.33	54.17	95.59	0.207
ΔFEV_1_% + ΔFEF_25–75_%	0.823	[0.751, 0.896]	72.86	90.48	65.31	52.78	94.12	0.575

The abbreviations are as defined for Tables 1, 2. Statistical significance is shown by bold font.

**FIGURE 2:**
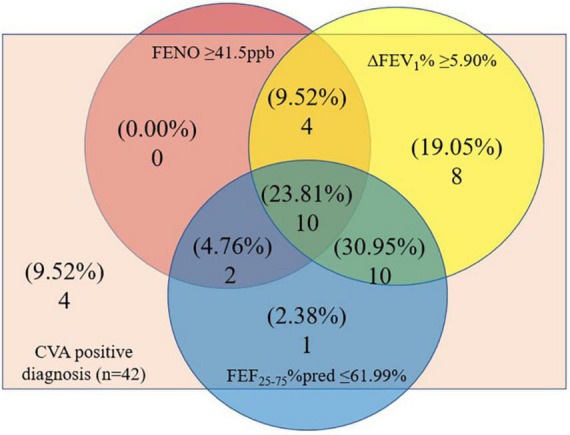
Venn diagram showing the overlaps of CVA positive diagnosis patients with ΔFEV_1_% ≥ 5.90%, FENO ≥ 41.5ppb, or FEF_25–75_%pred ≤ 61.99%. CVA, cough variant asthma; ΔFEV_1_%, the increase in forced expiratory in 1 s as a percentage of baseline value in a bronchodilation test; FEF_25–75_%pred, percentage of predicted forced expiratory flow at 25% to 75%; FENO, fractional exhaled nitric oxide.

Venn diagram was showing the overlaps of CVA positive diagnosis patients with FENO ≥ 41.50ppb, ΔFEV_1_% ≥ 5.90%, and FEF_25–75_%pred ≤ 61.99%. There were 32 (76.19%) patients in the CVA positive diagnosis group with ΔFEV_1_% ≥ 5.90%, 16 (38.1%) patients with FENO ≥ 41.50ppb, and 23 (54.76%) patients with FEF_25–75_%pred ≤ 61.99% (Figure 2).

### Clinical characteristics of the suspected group

In the CVA negative diagnosis group, according to the improvement of FEV_1_ and VAS, 34 patients were up to the standard of the suspected group. Baseline spirometric indices including FEV_1_%pred, PEF%pred, and FEFs%pred, in the suspected group were significantly higher than those in the CVA positive diagnosis group (*p* < 0.05). The increases in absolute including improvement-FEV_1_, FVC, FEF_25_, FEF_50_, FEF_75_, and FEF_25–75_, and percentage including improvement-FEV_1_%, FEF_25_%, FEF_50_%, and FEF_25–75_%, from baseline to the posttreatment in the suspected group were significantly higher than the other 64 patients in the negative group whose FEV_1_ increased by less than 200 ml and less than 12% (*p* < 0.05). Improvements in each spirometric index from baseline to after bronchodilation and treatment were shown in Table 4.

**TABLE 4:** Demographic data and clinical features of study participants in the negative group, suspected group, and CVA positive diagnosis group.

Characteristics and variables	Negative group (*n* = 64)	Suspicious group (*n* = 34)	CVA positive diagnosis group (*n* = 42)	*P*-value
Age (years)^¶^	**55.00 [24.75]**	**37.50 [23.25]^†^**	**42.00 [29.50]**	**0.002**
Gender, male (n,%)	**16 (25.00%)**	**18 (52.94%)**	**12 (28.57%)**	**0.015**
Height (cm)^¶^	**162.00 [9.50]**	**170.00 [16.25]^†^**	**165.00 [14.00]**	**0.021**
Weight (kg)^¶^	60.00 [10.50]	64.50 [16.50]	62.00 [19.25]	0.489
BMI (kg/m^2^)^¶^	22.86 [3.38]	22.18 [4.81]	22.81 [4.72]	0.439
Former smoker (n,%)	10(15.62%)	1(2.94%)	4 (9.52%)	0.148
Symptom duration (months)^¶^	7.50 [11.00]	6.50 [6.50]	9.50 [9.50]	0.21
Symptom recovery time (days)^¶^	**15.50 [22.75]**	**7.50 [12.50]**	**7.00 [5.50]** ※	**0.001**
VAS^¶^	**75.00 [13.80]**	**75.00 [15.00]**	**80.00 [10.00]** ^※#^	**0.005**
ΔVAS^¶^	**32.50 [55.00]**	**60.00 [21.30]**	**65.00 [16.30]** ※	**<0.001**
FENO (ppb)^¶^	**19.50 [17.00]**	**26.50 [23.00]**	**33.00 [32.25]** ※	**0.008**
WBC (*10^9^/L)^¶^	6.04 [2.78]	6.29 [2.37]	6.73 [2.06]	0.714
EOS%^¶^	2.00 [1.90]	1.80 [1.43]	1.95 [2.55]	0.817
EOS (*10^9^/L)^¶^	0.12 [0.13]	0.12 [0.08]	0.13 [0.20]	0.826
FVC%pred^¶^	**98.81 [16.18]**	**99.39 [11.23]**	**93.09 [14.30]** ※	**0.033**
FEV_1_%pred^¶^	**97.00 [14.78]**	**96.90 [17.09]**	**87.05 [17.42]** ^※#^	**0.001**
FEV_1_/FVC	0.82 ± 0.06	0.81 ± 0.06	0.79 ± 0.05	0.06
PEF%pred$	**97.97 ± 15.61**	**95.18 ± 11.54**	**86.33 ± 18.68** ^※#^	**0.001**
FEF_25_%pred$	**97.34 ± 19.76**	**92.81 ± 15.97**	**81.58 ± 18.00** ^※#^	**<0.001**
FEF_50_%pred$	**79.91 ± 22.96**	**80.45 ± 21.84**	**63.22 ± 14.19** ^※#^	**<0.001**
FEF_75_%pred	**62.01 [31.76]**	**65.60 [40.47]**	**49.96 [33.97]** ^※#^	**0.01**
FEF_25–75_%pred$	**74.55 ± 22.06**	**77.41 ± 21.99**	**59.63 ± 16.91** ^※#^	**<0.001**
Improvement-FVC	**115.00 [235.00]**	**230.00 [172.50]** ^†^	**390.00 [315.00]** ^※#^	**<0.001**
Improvement-FEV_1_	**95.00 [90.00]**	**280.00 [92.50] ^†^**	**480.00 [195.00]^※#^**	**<0.001**
Improvement- FEV_1_/FVC	**0.00 [0.05]**	**0.02 [0.03]** **^†^**	**0.05 [0.06]** ※	**<0.001**
Improvement-PEF	**-70.00 [1227.50]**	**150.00 [1365.00]**	**710.00 [1295.00]** ※	**<0.001**
Improvement-FEF_25_	**135.00 [1117.50]**	**485.00 [1050.00] ^†^**	**1140.00 [1610.00]** ^※#^	**<0.001**
Improvement-FEF_50_	**215.00 [552.50]**	**765.00 [615.00]^†^**	**930.00 [935.00]** ※	**<0.001**
Improvement-FEF_75_	**50.00 [460.00]**	**315.00 [377.50]^†^**	**410.00 [465.00]** ※	**<0.001**
Improvement-FEF_25–75_	**240.00 [515.00]**	**620.00 [480.00] ^†^**	**905.00 [672.50]** ※	**<0.001**
Improvement-FVC	**4.14 [7.31]**	**5.86 [4.66]**	**11.16 [9.14]** ^※#^	**<0.001**
Improvement-FEV_1_%	**3.97 [4.18]**	**9.46 [2.83] ^†^**	**18.00 [5.45]** ^※#^	**<0.001**
Improvement-PEF%	**-1.18 [22.02]**	**2.32 [17.92]**	**14.05 [20.54]** ^※#^	**<0.001**
Improvement-FEF_25_%	**1.18 ± 14.96**	**10.74 ± 14.44^†^**	**27.88 ± 25.39** ^※#^	**<0.001**
Improvement-FEF_50_%	**8.82 ± 18.26**	**24.20 ± 17.41^†^**	**39.26 ± 32.51** ^※#^	**<0.001**
Improvement-FEF_75_%	**5.86 [49.09]**	**14.30 [36.42]**	**45.73 [53.94] ^#^**	**<0.001**
Improvement-FEF_25–75_%	**8.45 [25.63]**	**22.24 [20.24] ^†^**	**48.41 [31.62]** ^※#^	**<0.001**

Improvement-spirometric indices, increase in spirometric indices from baseline to 4 weeks of anti-asthma treatment. **^†^**, the difference between the negative group and the suspected group was statistically significant; ※, the difference between the negative group and positive group was statistically significant; **^#^**, the difference between the suspected group and positive group was statistically significant. The other abbreviations are as defined in Tables 1, 2. Statistical significance is shown by bold font.

### Clinical characteristics of patients with symptom recover time ≤7 days and symptom recover time >7 days

There were 67 patients with symptom recovery time ≤7 days (47.86%), and 73 patients with symptom recovery time >7 days (52.14%). Patients with symptom recovery time ≤7 days in the positive group, the suspected group, and the negative group accounted for 69.05%, 50.00%, and 32.81%, respectively (Supplementary Figure 1). Patients with symptom recovery time ≤7 days had lower baseline spirometry, and higher spirometric improvement in BDR including the percentage change (ΔFEV_1_%) and absolute change (ΔFEV_1_). The improvement of spirometric after 4 weeks of treatment includes the percentage change (improvemet-FEV_1_%, FEF_25_%, FEF_25–75_%) and absolute change (improvement-FEV_1_, FEF_25_, FEF_75_, FEF_25–75_) in patients with symptom recover time ≤7 days was significantly higher than patients with symptom recover time >7 days. And patients with symptom recovery time ≤7 days had a higher baseline FENO (*p* = 0.011) and VAS score (*p* = 0.002) (Supplementary Table 1). We also collected the cough characteristic of these patients with symptom recovery time ≤7 days, most of them had fixed inducing factors and nocturnal or daytime cough, and the cough was commonly stimulating dry cough with average sputum less than 10 ml/day.

### Stratification analysis

Stratification analysis of baseline FEV_1_%pred divided the patients into 4 layers, and the percentage of ΔFEV_1_% > 12% was significantly different in the different subgroups. The percentage of the improvement of ΔFEV_1_% > 12% declined with increasing baseline FEV_1_%pred (*p* = 0.001) (Supplementary Figure 2A). However, the percentage of the improvement of ΔFEV_1_ > 200 ml had no significant correlation between baseline FEV_1_ stratification (*p* = 0.063) (Supplementary Figure 2B).

## Discussion

To our knowledge, our research pioneered in analyzing the spirometric changes in BDR to predict the diagnosis of CVA in patients with chronic cough and negative BDR. ΔFEV_1_% ≥ 5.90% for predicting the diagnosis of CVA and response to anti-asthma treatment may provide a simple, economical, and accessible method for primary care physicians without access to BCT. FEF_25–75_%pred alone or combined with FENO also had a predictive value for CVA diagnosis. In addition, patients with higher baseline FEV_1_%pred had a smaller percentage of FEV_1_ improvement after anti-asthma treatment, so clinical physicians might need to take baseline FEV_1_%pred into consideration when evaluating the result of BAAT.

Although most primary hospitals own spirometers, BCT is, to a large extent, hard to be performed. On top of that, many CVA patients have essentially normal baseline FEV_1_, leading to a low rate of positive BDR results. In our study, the baseline FEV_1_%pred of most patients was above 80%, but there were still some patients with the baseline FEV_1_%pred between 70 and 80%, whose BDR results were also negative. All these factors contribute to the seriously low diagnostic rate of CVA (14). Therefore, it is important to provide a simple and effective method to identify CVA patients timely, and predict the treatment response to diagnostic therapy.

Diagnostic therapy is helpful for early detection and effective treatment in CVA patients, which may prevent the development to classic asthma (4, 5). ICS is a fundamental medication for CVA treatment, and recent investigations suggest that combination therapy of ICS and bronchodilator provides more rapid and effective relief of cough symptoms than ICS or bronchodilator therapy alone (15, 16). Therefore, ICS combined with formoterol was used under medical supervision.

Through the diagnostic therapy, a total of 42 patients who met the BAAT-positive criteria were diagnosed with CVA. All spirometric indices including large and small airways in the CVA positive diagnosis group were lower than those in the CVA negative diagnosis group. Notably, despite the near-normal large airway function in CVA patients, small airway dysfunction was remarkable, which was consistent with our previous study (17, 18). We also found a lower FEV_1_/FVC in the CVA positive diagnosis group, which indicated an expiatory airflow limitation according to GINA.

Some studies have shown that FENO has a certain value in predicting the diagnosis of CVA, but its independent diagnostic value remains controversial (19–21). In our study, when FENO was used alone for predicting CVA, the AUC was significantly lower than ΔFEV_1_%, indicating that FENO alone is insufficient for predicting CVA in patients with chronic cough. When combined with ΔFEV_1_%, its predictive accuracy improved but was not significantly higher than ΔFEV_1_% alone. Compared with FENO, we recommend routine BDR in primary care settings to help diagnose CVA, and the FENO test might be further performed if conditions permit.

Small-airway indices such as FEF_25–75_%pred alone or combined with FENO are effective in predicting CVA according to our previous research (22, 23). FEF_25–75_%pred also had a good predictive value for the diagnosis of CVA in this study, but compared with ΔFEV_1_%, it was lower. The different results of predicted models were likely due to the different evaluation criteria of the CVA diagnosis, the former was BCT and now was the response to anti-asthma treatment. In terms of predicting the effect of anti-asthma treatment, the predictive value of ΔFEV_1_% in BDR was higher than that of FEF_25–75_%pred. However, the Venn diagram showed that some patients with CVA were identified by FEF_25–75_%pred dysfunction rather than ΔFEV_1_% in BDR, indicating small airway dysfunction was also important in predicting the diagnosis of CVA and therapeutic response. Therefore, we recommended that small-airway indices should be conducted at the same time, because it may help to furtherly improve the diagnostic rate of CVA.

Patients with symptom recovery time ≤7 days may suffer from corticosteroid-responsive cough (CRC), which includes cough variant asthma, eosinophilic bronchitis, and atopic cough (24). Patients with the shorter symptom recovery time had a higher FENO and VAS at the baseline, and also had higher spirometric improvement in BDR after 4 weeks of treatment, which suggested a higher airway inflammation and well response to ICS + LABA. Furthermore, symptom recovery time ≤7 days may be a signal that the anti-asthma treatment will be effective. Therefore, patients in primary care with a stimulating dry cough in nocturnal or daytime, high FENO, and even no significant improvement in BDR should equally be considered a treatment with ICS and evaluated the response within 7 days.

The absolute change of FEV_1_ from baseline to 4 weeks of treatment in the suspected group was significantly greater than that in the negative group (ΔFEV_1_ ≤ 200 ml and ΔFEV_1_% ≤ 12%). But its baseline FEV_1_ was significantly higher than that in the CVA positive diagnosis group. It is worth mentioning that the percentage change of FEV_1_ is negatively correlated with baseline FEV_1_, which may cause difficulty for patients in the suspected group to meet the positive criteria of BAAT even if the absolute change is much more than 200 ml. Nevertheless, our results warrant further investigations to determine whether this group of patients has a limited percentage of improvement due to high baseline FEV_1_ or other diseases such as eosinophilic bronchitis that ICS was effective but the improvement of spirometry was limited.

Identically, an influence of baseline FEV_1_ on BAAT outcome was observed. By stratifying baseline FEV_1_, we found that the higher the baseline FEV_1_, the fewer patients achieved an improvement of FEV_1_% > 12% after anti-asthma treatment. Therefore, CVA may not be ruled out for those patients with high baseline FEV_1_ even if they didn’t meet the BAAT-positive criteria. In these scenarios, BCT should be performed to further confirm the diagnosis. If without access to BCT, it could be inferred by the response time of the treatment, because the symptom recovery time of the CVA positive diagnosis group and suspected group was both about 7 days. Meanwhile, there was no significant correlation between baseline FEV_1_ stratification and patients with an absolute change of FEV_1_ > 200 ml after anti-asthma treatment. Whether an improvement in FEV_1_ > 200 ml can be considered alone as a positive criterion for BAAT diagnosis of CVA with high baseline FEV_1_ requires further clinical research to verify.

It is important to recognize some limitations of our study. First, the study is single-centered with a relatively small sample size, therefore multicenter, large-scale, prospective studies may be needed in the future to further validate the results. Second, patients in the suspected group will need further observations to make a definitive diagnosis. Third, due to the limited availability of the cough monitoring system, we used patient-reported outcomes rather than objective cough measures. Fourth, due to lack of dynamic laryngoscopy, vocal cord dysfunction was rule out by spirometry with a low and straight inspiratory phase curve, which may result a mixed in this study.

In conclusion, our study proposed that ΔFEV_1_% ≥5.90% in BDR can be used to predict the diagnosis of CVA and the response of anti-asthma treatment in patients suffering from a chronic cough with negative BDR. In primary care settings without access to BCT, BDR is a simple and economical method to help physicians improve the diagnosis accuracy of CVA and help distinguish which patients should receive diagnostic trials of anti-asthma treatment. For chronic cough patients with high baseline FEV_1_%pred and significant improvement of the absolute value of FEV_1_ after anti-asthma treatment, the diagnosis of CVA may not be completely ruled out even if the positive criteria of BAAT are not met. While it is still premature to make any assumptions, physicians need to consider this new perspective when making personalized diagnoses and treatment plans for patients with chronic cough.

## Data availability statement

The raw data supporting the conclusions of this article will be made available by the authors, without undue reservation.

## Ethics statement

The studies involving human participants were reviewed and approved by the Institutional Review Board at Shanghai General Hospital (no. [2020]30). The patients/participants provided their written informed consent to participate in this study.

## Author contributions

MZ and HH conceived of and designed the entire study. YZ, WB, YX, and JL contributed to the data collection. CL performed spirometry, BDT, and FENO. YZ and MZ were involved in interpreting the clinical data. HH and ZeX performed statistical analyses. HH, ZiX, and YP wrote the manuscript and supervised by MZ. All authors critically reviewed and approved the final version and agreed to be accountable for all aspects of the work in ensuring that questions related to the accuracy or integrity of any part of the work are appropriately investigated and resolved.

## Supplementary material

Supplementary Figure 1 Symptom recovery time in the positive group, suspected group, and negative group.

Supplementary Figure 2 The improvement of FEV_1_ from baseline to 4 weeks of anti-asthma treatment in different layers of FEV_1_%pred. FEV_1_%pred, percentage of predicted forced expiratory in 1 s.
