# Supplementary figures and images for: Prediction of bronchodilation test in adults with chronic cough suspected of cough variant asthma
Source: Front Med (Lausanne). 2022 Dec 9;9:987887. doi: 10.3389/fmed.2022.987887 (PMC9780531; doi:10.3389/fmed.2022.987887)

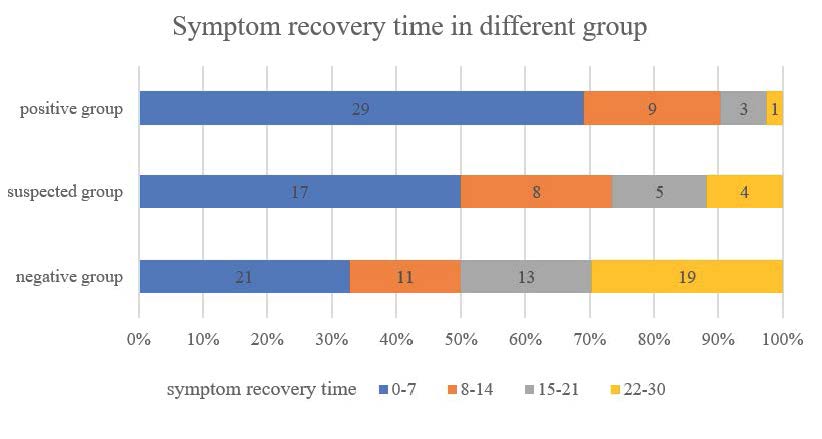

Supplement: Supplementary Figure 1 — Symptom recovery time in the positive group, suspected group, and negative group. [file Image_1.JPEG]

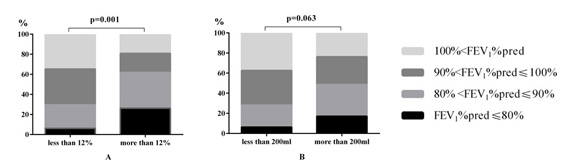

Supplement: Supplementary Figure 2 — The improvement of FEV1 from baseline to 4 weeks of anti-asthma treatment in different layers of FEV1%pred. FEV1%pred, percentage of predicted forced expiratory in 1 s. [file Image_2.JPEG]
