# Supplementary material for: Prediction of bronchodilation test in adults with chronic cough suspected of cough variant asthma
Source: Front Med (Lausanne). 2022 Dec 9;9:987887. doi: 10.3389/fmed.2022.987887 (PMC9780531; doi:10.3389/fmed.2022.987887)
Supplement: Supplementary file 3 [file Table_1.docx]

**TABLE E1.** Demographic data and clinical features of participants with different symptom recovery time.

| Characteristics and variables | Symptom recovery time ≤7 days (n=67) | Symptom recovery time >7 days (n=73) | p value |
| --- | --- | --- | --- |
| Age(years) ¶ | 41.00 [29.00] | 50.00 [26.50] | 0.075 |
| Gender, male (n, %) | 24 (35.82%) | 22 (30.14%) | 0.474 |
| BMI (kg/m^2^) § | 22.76 ± 10.70 | 22.78 ± 10.63 | 0.982 |
| Symptom duration(months) ¶ | 8.00 [12.00] | 8.00 [9.00] | 0.742 |
| VAS_1_¶ | **80.00 [10.00]** | **70.00 [15.00]** | **0.002** |
| ΔVAS¶ | **65.00 [25.00]** | **45.00 [50.00]** | **<0.001** |
| FENO(ppb) ¶ | **29.00 [29.00]** | **20.00 [20.50]** | **0.011** |
| WBC(*10^9^/L) ¶ | 6.43 [1.91] | 6.07 [2.61] | 0.451 |
| EOS%¶ | 1.90 [2.20] | 1.90 [1.80] | 0.764 |
| EOS(*10^9^/L) ¶ | 0.13 [0.18] | 0.12 [0.13] | 0.352 |
| FVC%pred§ | 97.18 ± 12.05 | 99.98 ± 11.69 | 0.175 |
| FEV_1_%pred§ | 92.65 ± 15.05 | 96.45 ± 16.90 | 0.06 |
| PEF%pred§ | **89.79 ± 17.30** | **97.48 ± 20.05** | **0.005** |
| FEF_25_%pred§ | **85.96 ± 21.69** | **96.61 ± 21.75** | **0.001** |
| FEF_50_%pred¶ | 68.24 [28.79] | 75.28 [33.06] | 0.256 |
| FEF_75_%pred¶ | 57.34 [32.64] | 61.63 [33.20] | 0.527 |
| ∆FVC¶ | 70.00 [150.00] | 60.00 [140.00] | 0.084 |
| ∆FEV_1_¶ | **160.00 [150.00]** | **110.00 [145.00]** | **0.008** |
| ∆PEF¶ | 290.00 [920.00] | 250.00 [705.00] | 0.301 |
| ∆FEF_25_¶ | 380.00 [830.00] | 350.00 [890.00] | 0.482 |
| ∆FEF_50_¶ | 360.00 [420.00] | 420.00 [615.00] | 0.859 |
| ∆FEF_75_¶ | 240.00 [300.00] | 150.00 [285.00] | 0.094 |
| ∆FEF_25-75_¶ | 410.00 [300.00] | 310.00 [475.00] | 0.380 |
| ∆FVC%¶ | 2.15 [3.92] | 1.39 [4.48] | 0.154 |
| ∆FEV_1_%¶ | **6.02 [5.88]** | **5.12 [4.85]** | **0.009** |
| ∆PEF%¶ | 4.18 [12.75] | 4.05 [12.80] | 0.346 |
| ∆FEF_25_%¶ | 6.98 [16.37] | 5.68 [14.81] | 0.530 |
| ∆FEF_50_%¶ | 11.90 [21.74] | 14.46 [23.34] | 0.846 |
| ∆FEF_75_%¶ | 27.37 [35.17] | 15.63 [33.86] | 0.095 |
| ∆FEF_25-75_%¶ | 16.61 [19.43] | 15.59 [21.97] | 0.485 |
| FEF_25-75_%pred¶ | 64.84 [24.72] | 70.24 [31.49] | 0.292 |
| improvement-FVC¶ | 250.00 [290.00] | 170.00 [200.00] | 0.057 |
| improvement-FEV_1_¶ | **320.00 [320.00]** | **160.00 [195.00]** | **<0.001** |
| improvement-PEF¶ | 500.00 [1410.00] | 100.00 [1390.00] | 0.09 |
| improvement-FEF_25_¶ | **750.00 [1220.00]** | **270.00 [1305.00]** | **0.008** |
| improvement-FEF_50_¶ | 640.00 [930.00] | 450.00 [800.00] | 0.176 |
| improvement-FEF_75_¶ | **320.00 [530.00]** | **130.00 [485.00]** | **0.041** |
| improvement-FEF_25-75_¶ | **640.00 [700.00]** | **350.00 [760.00]** | **0.019** |
| improvement-FVC%¶ | 7.91 [8.76] | 5.57 [6.13] | 0.156 |
| improvement-FEV_1_%¶ | **10.78 [11.59]** | **6.10 [7.67]** | **<0.001** |
| improvement-PEF%¶ | 7.23 [25.28] | 2.16 [21.29] | 0.073 |
| improvement-FEF_25_%¶ | **12.22 [23.77]** | **4.25 [24.00]** | **0.006** |
| improvement-FEF_50_%¶ | 21.43 [32.79] | 14.84 [28.16] | 0.184 |
| improvement-FEF_75_%¶ | 33.88 [58.10] | 10.19 [57.98] | 0.061 |
| improvement-FEF_25-75_%¶ | **28.21 [34.20]** | **17.01 [33.23]** | **0.029** |

FENO, fractional exhaled nitric oxide; VAS_1_, visual analog scale score at the first visit; ΔVAS, improvement of VAS from baseline to 4 weeks of treatment; WBC, white blood cells; EOS, eosinophils; BMI, body mass index; FVC, forced vital capacity; FEV_1_, forced expiratory volume in one second; PEF, peak expiratory flow; FEF_25_, forced expiratory flow at 25% of forced vital capacity; FEF_50_, forced expiratory flow at 50% of forced vital capacity; FEF_75_, forced expiratory flow at 75% of forced vital capacity; FEF_25-75_, forced expiratory flow at 25% to 75% of forced vital capacity; %pred, the actual measured value of spirometric indices as a percentage of the predicted value. ∆, increase in spirometric indices in BDR; Δ%, spirometric indices%, increase in spirometric indices as a percentage of the baseline value. improvement-spirometric indices, increase in spirometric indices from baseline to 4 weeks of anti-asthma treatment. § Mean ± standard deviation values; ¶ Median [IQR] values; Statistical significance is shown by bold font.
